# mSphere of Influence: Embracing biological complexity

**DOI:** 10.1128/msphere.00218-26

**Published:** 2026-08-17

**Authors:** Linda Catherine Horianopoulos

**Affiliations:** 1 Department of Food Science, University of Guelph 3653, Guelph, Canada; University of Guelph, Guelph, Ontario, Canada

**Keywords:** yeast biology, systems biology, evolutionary biology, functional genomics

## Abstract

Linda Horianopoulos is a yeast biologist who uses a combination of comparative genomics, evolutionary analyses, and targeted molecular genetics to research the diversity of metabolism and stress responses across different yeast species. In this mSphere of Influence article, she highlights how a research article about Hsp90’s ability to potentiate rapid evolution of drug resistance across different fungal species (https://doi.org/10.1126/science.1118370) influenced her work and the lens through which she appreciates biological complexity.

## COMMENTARY

Sometimes a specific research topic can inspire a different way of thinking that sticks with you well beyond the project itself. When I began my PhD on the roles of Hsp40 co-chaperones in the pathogenesis of *Cryptococcus neoformans,* I was inspired to learn that chaperone biology was an area strongly influenced by female scientists who pioneered the molecular characterization of heat shock proteins (HSPs) in yeast (1, 2). I became engrossed by the molecular chaperone and HSP literature. The early work in co-chaperones was detailed and highly mechanistic. This really appealed to me as I started my project because I loved the idea that I could make targeted genetic manipulations and understand the effect of specific co-chaperones on *C. neoformans* biology and pathogenesis.

I was reading background literature on the role of chaperones in fungal pathogens when I came across a 2005 *Science* paper by Leah Cowen and Susan Lindquist. They described the role of a core heat shock protein (Hsp90) in the acquisition of drug resistance in the model yeast, *Saccharomyces cerevisiae* (3). This paper also extended their findings to pathogenic fungi, showing that Hsp90 has broad impacts on the resistance to antifungal drugs. However, the exact details varied across species, since different fungi needed Hsp90 to acquire resistance to different antifungal drugs depending on their downstream signaling pathways. For instance, in *Candida albicans,* Hsp90 promotes resistance against fluconazole, whereas in *Aspergillus terreus*, it promotes resistance against caspofungin. In both cases, inhibitors of the phosphatase calcineurin, which is also a Hsp90 client protein, phenocopy the inhibitors of Hsp90, suggesting that the impact on different drugs is reflective of divergent signaling pathways in each species. Overall, this illustrates how evolution can repurpose core pathways to achieve distinct outcomes depending on the life history strategies of each organism.

The interconnected nature of chaperones and signaling pathways also pushed me to think beyond individual genes or proteins and toward the networks in which they operate. In studying Hsp40 co-chaperones, it became clear to me that their contributions to pathogenesis could not be understood in isolation. Their impact depended on the availability of client proteins, interactions with core chaperones, such as Hsp70 or Hsp90, and coordination with other components of the proteostasis machinery. Mutating or deleting a single co-chaperone was not simply the loss of one function, but a disruption of multiple interconnected pathways. This challenged the classical “one gene, one phenotype” framework and encouraged a more systems-level view of fungal biology. As a result, I tested more phenotypes to look for indirect effects, global changes, and genetic interactions, expanding the breadth and impact of my research.

After finishing my PhD, I kept the lens of comparative genomics at the forefront of my approach. I wanted to build on what I had learned about stress responses focused on a single species and push myself to learn more about comparative genomics and evolutionary biology in multiple species. So, I joined Chris Todd Hittinger’s lab for my postdoctoral research. The Hittinger and Rokas labs had recently published the genomes of 332 yeast species (4) and were actively working on sequencing nearly 800 more (5). This presented a fantastic opportunity to explore interspecific variation in cellular stress and use genomic data to understand the evolutionary mechanisms driving this variation. We could explore questions such as which processes are conserved across hundreds of millions of years of evolution and which have arisen convergently through distinct molecular mechanisms (6). We observed that even deeply conserved genes and pathways can produce different outcomes depending on their evolutionary context.

In developing my independent research program, I keep the 2005 Cowen and Lindquist paper in mind as one of those illustrative stories about the complexity of biological systems. It highlights why it is important to study diverse organisms to understand what is conserved and what varies. As an early career researcher at a time when large data sets are readily available and AI can make rapid predictions, this lesson is especially relevant. Many predictions are extrapolated from a limited number of studies in model organisms, yet even highly conserved genes can function differently in the context of non-model species. I hope that embracing biological complexity can help my group to uncover some of the surprising aspects that drive diverse phenotypes across yeasts.
